# Depression Prior to Dementia: Examining Its Role as a Risk Factor, Prodromal Marker, or Confounding Comorbidity: A Synthesis of Current Research

**DOI:** 10.1192/bjo.2025.10126

**Published:** 2025-06-20

**Authors:** Virginia Johnson, Calvin Flowers

**Affiliations:** CFMD Inc, Visalia, USA

## Abstract

**Aims:** The relationship between depression and dementia represents a complex clinical phenomenon that continues to challenge our understanding of neurodegenerative disease progression. This review synthesizes the most recent evidence examining whether depression serves as a risk factor, prodromal marker, or common confounding comorbidity in dementia development.

**Methods:** A comprehensive review of recent research studies analysing the psychiatric markers associated with dementia onset were reviewed to develop a clinical framework for understanding and analysing the degree to which these psychiatric phenotypes are representing either risk factors, prodromal psychiatric markers or simply overlapping psychiatric comorbidity.

**Results:** Recent longitudinal research has revealed that mental disorders, particularly depression, significantly increase dementia risk, with symptoms manifesting up to two decades before dementia diagnosis. This research demonstrated that depressive symptoms often emerge as early as 15 years before formal dementia diagnosis, suggesting its potential role as a prodromal marker. These findings align with recent meta-analytic evidence confirming depression as an independent risk factor for dementia development.

Research has identified specific inflammatory pathways linking depression and neurodegeneration, with elevated inflammatory markers serving as a potential biological bridge between these conditions. This neuroinflammatory process appears to be bidirectional, with depression potentially increasing inflammatory markers that may accelerate cognitive decline, while neurodegenerative processes can trigger inflammatory responses that exacerbate depressive symptoms. These biological markers suggest shared pathophysiological pathways between depression and neurodegenerative processes, with inflammation playing a central role in both conditions.

**Conclusion:** The synthesis of research findings has significant implications for understanding and developing appropriate clinical practice and preventive strategies. The identification of depression as a risk factor, confounding variable and potential prodromal marker, is generally supported by robust longitudinal evidence and biological mechanisms and emphasizes the need for early intervention and regular monitoring of cognitive function in individuals with late-life depression. The evidence suggests a complex multifactorial interplay where depression may serve as a risk factor, comorbidity and an early manifestation of neurodegenerative processes, highlighting the importance of comprehensive assessment and long-term monitoring of depressed elderly patients for cognitive decline.

